# Characterizing Emergency Department Disposition Conversations for Persons Living With Dementia: Protocol for an Ethnographic Study

**DOI:** 10.2196/65043

**Published:** 2024-12-06

**Authors:** Justine Seidenfeld, Matthew Tucker, Melissa Harris-Gersten, Gemmae M Fix, Ivonne Guzman, Nina R Sperber, Susan N Hastings

**Affiliations:** 1 Center of Innovation to Accelerate Discovery and Practice Transformation (ADAPT) Durham VA Health Care System Durham, NC United States; 2 Department of Emergency Medicine Duke University School of Medicine Durham, NC United States; 3 Department of Emergency Medicine Durham VA Health Care System Durham, NC United States; 4 Duke University School of Nursing Durham, NC United States; 5 Center for Health Optimization and Implementation Research VA Bedford Healthcare System Bedford, MA United States; 6 Section of General Internal Medicine Boston University Chobanian & Avedisian School of Medicine Boston, MA United States; 7 Department of Population Health Sciences Duke University School of Medicine Durham, NC United States; 8 Division of Geriatrics Department of Internal Medicine Duke University School of Medicine Durham, NC United States

**Keywords:** ethnography, direct observations, emergency medicine, dementia, caregivers

## Abstract

**Background:**

Almost 40% of persons living with dementia make an emergency department (ED) visit each year. One of the most impactful and costly elements of their ED care is the decision to discharge or admit them to the hospital—the “disposition” decision. When more than one reasonable option exists regarding a health care decision, such as the decision to admit or not, it often requires a complex conversation between patients, care partners, and ED providers, ideally involving shared decision-making. However, little is known about how these conversations are conducted and the real-world context in which they take place. Best practices in ED communication and shared decision-making for persons living with dementia and their care partners are limited.

**Objective:**

This study aims to characterize current practices in ED disposition conversations for persons living with dementia and their care partners, informed by perspectives from patient and care partner participants.

**Methods:**

This study will use an ethnographic design, including direct observation methods with a semistructured data collection tool to capture the ED encounter for up to 20 patient and care partner dyads, including all discussions about dispositions. Follow-up qualitative, semistructured interviews will be conducted with persons living with dementia and their care partners to explore specific observations made during their ED encounter, and to gain insight into their perspective on their role and elements of decision support used during that conversation.

**Results:**

Data collection was initiated in October 2023, with 13 dyads recruited and observed as of July 2024. This study is expected to be completed by December 2024.

**Conclusions:**

Novel methods can offer novel insights. By combining direct observation and follow-up interviews about an ED visit, our study design will provide insights into how ED disposition occurs in real-world settings for persons living with dementia. Findings can inform more patient-centered interventions for disposition decision-making.

**International Registered Report Identifier (IRRID):**

DERR1-10.2196/65043

## Introduction

### Background

For persons living with dementia, emergency department (ED) visits often represent a sentinel event, with a significant risk of subsequent increased health care utilization and long-term care placement [1,2]. About 40% of the estimated 2.4-5.5 million US population living with dementia have an ED visit each year [3]. ED care for persons living with dementia can be challenging because these patients have more comorbidities and medications compared with their counterparts without dementia, and they frequently find the ED environment overstimulating and disorienting [4]. In many cases, they may not be able to provide a clinically actionable account of what brought them to the ED, in which case ED providers often rely on collateral information from care partners or anyone available.

Among the many decisions that are made over the course of an ED visit, one of the most difficult is the disposition decision; ED providers must balance the risks associated with hospital admission, including delirium and functional decline, against the risks of discharge, which may result in return visits to the ED and hospitalizations [5]. This decision has enormous implications for patient experience, outcomes, and costs of care and is especially intricate for persons living with dementia, as they have a higher risk of these adverse events compared with their counterparts without dementia [6].

In ED visits when this choice is not straightforward, a high-quality disposition decision would ideally involve input from the person living with dementia and their care partner through shared decision-making (SDM) [7]. While SDM is more widely used in other clinical contexts, it is a relatively new but growing concept in the ED setting, with great potential to ensure that ED decisions reflect the goals, preferences, and values of patients and their care partners [8,9]. Currently, there are no best practices or SDM tools to help guide these complex ED disposition conversations.

However, effective interventions to address the lack of best practices in SDM in this context will require real-world data about the context within which they are implemented, and we lack a fundamental understanding of how these conversations currently take place [10]. For example, to determine the optimal timing for an SDM tool, we need foundational data on current communication practices for disposition discussions (eg, is it addressed across multiple interactions over the course of an ED visit, or mostly at the very end?) [11]. To best facilitate participation from persons living with dementia and care partners, we need to understand what kind of decisional roles they each tend to take. To improve the degree of “sharing” during these discussions, we need to understand barriers and facilitators to participating in an SDM conversation around disposition (eg, are they invited to share their relevant values or preferences, is there sufficient time to make a decision?).

To address these gaps, the primary objective of this study is to use ethnographic methods to characterize current practices in disposition conversations for older persons living with dementia [12]. This will be augmented by follow-up interviews with patients and care partner participants to elicit their perspectives on observed barriers and facilitators to participation in these conversations. Direct observation methods are uncommon in emergency medicine research but can provide unique and nuanced information about health care processes and behavior that may not be captured by self-reported means such as surveys or participant recall in interviews [13]. It can facilitate researchers’ understanding of how patients experience a specific event or circumstance. A previous scoping review on communication and SDM for persons living with dementia in the ED demonstrated regular use of interviews, focus groups, and survey methods [14]. To our knowledge, this will be the first use of direct observation methods with persons living with dementia in the ED setting.

### Research Aims and Objectives

#### Aim 1

The first aim is to characterize discussions about ED disposition with persons living with dementia and their care partners using direct observation methods during ED visits, focusing on timing, decisional roles, and decision support strategies used in disposition conversations.

#### Aim 2

The second aim is to identify facilitators and barriers to participating in disposition decision-making for persons living with dementia and care partners. Informed by findings from direct observations in aim 1, we will conduct semistructured qualitative interviews with aim 1 participants. Interviews will ask participants about specific observations made from their encounter, to gain insight into their perspective on their role and elements of decision support used during that conversation.

## Methods

### Study Design

#### Overview

This ethnographic study design entails 2 largely concurrent, complementary phases [15]. Patient and care partner dyads will be recruited at the time of an ED visit, with direct observations using a semistructured data collection tool done during the ED visit that day. Follow-up interviews will be conducted within 48-72 hours of the observations, albeit dependent on the status of the patient given their health care needs and care partner availability.

This research plan is informed by the Ottawa Decision Support Framework (ODSF), a conceptual framework of decision-making commonly used in the development of patient decision aids [16,17]. The ODSF is guided by the premise that there are unmet decisional needs (eg, decisional conflict and unclear values) that, when addressed with “decision support,” will improve decisional outcomes. As the focus of this proposal is to better understand current practices in decision support and sharing in decision-making, our study will be structured around elements that play a role in decision support in the ODSF.

#### Setting and Context

Data will be collected at the Durham Veterans Affairs Medical Center (DVAMC), a 251-bed tertiary care referral, teaching, and research facility affiliated with Duke University School of Medicine in Durham, North Carolina. The DVAMC ED evaluates and treats approximately 35,000 patients annually. The DVAMC ED achieved level 1 status (the highest level) of Geriatric ED accreditation from the American College of Emergency Physicians in June 2023 [18]. Of note, the DVAMC is also an Age Friendly Health System and working toward Geriatric Surgery Verification [19].

#### Researcher Characteristics and Reflexivity

Two researchers will screen patients for recruitment and perform data collection and analysis in this study. JS is a practicing emergency medicine physician at the DVAMC and a Health Services Researcher and brings an understanding of workflow and procedures in the DVAMC ED. MT is a qualitative data analyst in Health Services Research with a background in anthropology. Given the study design requiring immediate observational data collection after participant recruitment, the researchers also perform real-time screening in the ED and approach patients for consent before data collection.

#### Sampling Strategy

Inclusion criteria for patient and care partners include the following: (1) the patient is aged 65 years or older; (2) there is a dementia diagnosis documented in the electronic medical record (EMR); (3) the patient is community dwelling; (4) the patient or care partner (depending on the severity of a patient’s dementia and if the patient has a legally authorized representative) is able to discuss disposition in English; and (5) the patient does not have a straightforward disposition based on study principal investigator–clinician review of initial triage note and vitals. Exclusion criteria include the following: (1) the patient has no care partner available in the ED; (2) the patient is triaged as Emergency Severity Index Level 1 (indicating a high-acuity patient that is very likely to be admitted); (3) the patient is not community dwelling; and (4) the patient has medical instability, acute altered mental status, or hyperactive delirium at the time of the ED visit (similarly indicative of a patient that is likely to be admitted). ED health care providers included may be attendings and residents, and any provider may be observed for multiple encounters if they care for more than one participant.

#### Recruitment Procedures

Trained observers will be stationed in the DVAMC ED for study recruitment. Eligible veterans and care partner dyads will be identified at the time of triage or check-in through monitoring the DVAMC electronic ED track board. Potentially eligible participants will first be identified using the EMR for inclusion and exclusion criteria. A documented diagnosis of dementia can be identified through several means, including International Classification of Diseases codes assigned to the patient’s record, documentation in previous notes including outpatient visits or discharge summaries, or using the EMR search function with the query “dementia.” These strategies were selected as those that best replicate the means by which ED providers often identify persons living with dementia in the ED clinical environment. For eligible participants, the study principal investigator will be contacted by the research staff to clinically review the nursing triage note and vitals as soon as they are available and make a determination as to whether the ED visit is likely to warrant deliberation about disposition by the average ED provider. Patients or care partners will not be approached before being placed in an ED room or while in the ED waiting room, given privacy concerns. When a potentially eligible patient has been assigned to an ED room on the electronic ED track board, the researchers will speak to the ED physician providers who will most likely care for that patient (based on room assignment location) to ask if they would be willing to delay their initial conversation for our consent process. If there are any concerns about this delay, we defer consent to allow for appropriate patient care. Of note, our protocol excludes any patients who are unstable or acutely agitated and thereby ensures that the time required for consent with the patient and care partner does not delay any crucial timely ED care. The patient and care partner are then approached to participate in the study after they have been placed in a private ED room.

#### Consenting Procedures

When the patient and care partner dyad are approached by the researcher, they are given a flyer that explains the study and asked if they would be interested in learning more about the study while they wait for a provider. With the targeted population of persons living with dementia, including those with moderate to severe dementia, their capacity to consent to research may significantly vary, although the severity of their dementia may not directly correspond to their capacity [20]. However, if this study was limited only to patients who had the capacity to provide consent, there is a significant risk that this would make the study population less representative. We acknowledge that persons living with dementia are a vulnerable population, and we will assess prospective participants’ capacity to consent using the Evaluation to Sign Consent Scale, a measure developed to evaluate nursing home residents’ ability to communicate and provide informed consent [21]. Of note, if the care partner at the bedside at the time of the ED visit is not the highest priority surrogate decision maker for the patient, the highest priority surrogate available at the time will be contacted for consent for the patient to participate, and the care partner at the bedside will additionally be consented for their own participation. If the person living with dementia is unable to fully appreciate key features of the protocol, the potential risks, or their rights to withdraw, we will ask the participant if they would like their highest priority surrogate decision maker or legally authorized representative (LAR) to provide consent. The person living with dementia will still provide assent to study staff explaining the procedures involved in the protocol in simplified terms. To respect the dignity of the participants living with dementia, we will also respect their dissent (which can include low levels of cooperation, demonstrating frustration, or passivity) in combination with proxy consent.

All participants that consent to participate in direct observations will be asked if they are also willing to participate in follow-up interviews, and we will additionally acquire their consent through written consent at that time. If any signs of discomfort are exhibited by the person living with dementia or it is requested, the researchers will stop the research process at that time.

### Ethical Considerations

This research received approval from the DVAMC institutional review board (approval #1820207) in September 2023. For participating in this study, participants will receive US $50 for observations during the ED encounter and an additional US $50 for participating in follow-up interviews. While no identifying information is collected for the ED health professionals observed, written consent is also collected for them.

### Data Collection

#### Direct Observations

The unit of observation is an interaction between the provider and the patient or care partner. Anticipating that each ED visit will include multiple discrete interactions between the patient or care partner, we will use continuous event sampling to collect data, meaning that all interactions are sequentially recorded during the ED visit [22]. Data will be collected from the time point of the first interaction after the patient has been placed in an ED room up until the point that a disposition decision is made. Observers will function as a “complete observer,” meaning that the researcher observes what is occurring without interfering in routine care [22].

Data collection will occur through mobile positioning, in which the observer follows a person during a given activity or for a particular observation period [22,23]. As such, we will collect data as the patient and care partner move throughout the ED. The observers will station themselves in as unobtrusive a location as possible, ensuring that persons living with dementia and care partners are comfortable with their position. The data collection tool is a paper-based semistructured template, selected as it was thought to be less intrusive than typing on a keyboard if using an electronic form during an observation. Data will include the (1) date, (2) time, (3) location, (4) people present, (5) duration, and (6) contents of the discussions. Discussions will be captured using an open section for descriptive field notes. Descriptive fieldnotes can capture general observations, nonverbal communication such as facial expressions and demeanor, as well as paraphrased statements from the participants [12,24]. These notes will include topics such as who the physician is addressing during the conversation, the medical history of the patient, the reason for visit, and all components of the disposition discussion. Any interpretive notes (eg, that the care partner seems frustrated) will be clearly identified with “[closed brackets]” to demarcate this from the descriptive data. This approach leaves room for unanticipated findings or documentation of activities that are otherwise uncategorized. While the data collected during the encounter itself may be brief, the observer will fill in additional detail or add more thorough information after the encounter is over, with all notes written by the end of the day to minimize recall bias. Researchers will also write a postobservation reflection to summarize their overall impression of the ED encounter by the end of the day. Multimedia Appendix 1 presents the data collection tool template.

#### Baseline Data Collection

During a portion of the ED visit during which ED providers are not present, and no active patient care is taking place (eg, while waiting for test results), we will collect additional features of patients and care partners that may influence disposition decisions, including dementia severity by using the 5-minute, informant-based Dementia Severity Rating Scale [25,26]. To further describe the study sample, we will also measure patient demographics, income, health literacy through the Short Literacy Survey [27], and care partner burden (Zarit Burden Interview 4-item) [28]. Care partners will be asked to fill out the latter directly, instead of by interview.

#### Semistructured Interviews

Follow-up semistructured interviews will be conducted by the researcher who collected the direct observation data whenever possible. These will be scheduled at the time of the ED visit for participants who are discharged home. For participants who are admitted, a time to contact the care partner will be established, at which time the follow-up interviews can be scheduled. We aim to conduct 30- to 45-minute interviews with care partners and patients as much as possible. Interviews will be audio recorded and transcribed. For persons living with dementia, questions will be simple, with fewer planned questions and more probes to elicit details. Interview guides are structured around elements of decision support observed in aim 1. We may use specific observations and aim 1 results as prompts to elicit discussion and to gain insight into their perspective on their role and elements of decision support used. Multimedia Appendix 2 presents the interview guide templates.

### Data Analysis

#### Overview

Our analytic goal is to characterize discussions about ED dispositions with persons living with dementia and their care partners, and to describe how different elements of SDM are used, informed by participant perspectives. Each participant unit will consist of a person living with dementia and their care partner, and for each of those, one unit of data will consist of one direct observation template and fieldnotes, 1 or 2 interview transcripts, and baseline demographic and clinical survey data. Data will be analyzed by the same 2 researchers performing data collection.

Data analysis for direct observations will be conducted using the constant comparative method, in which data collection and analysis are interrelated and carried out concurrently [29]. An initial codebook for each unit of data (encompassing the direct observation and interview data) was developed based on elements of SDM outlined in the ODSF and the research question. Analysts will independently code a minimum of 2 units of data and will refine the codebook based on findings. Postobservation reflections will also guide the development of the codebook. Data analysis will use iterative rounds of more focused coding to organize the initial coding results into themes. The data source (ie, direct observation notes, the patient interview, or the care partner interview) for each piece of data will be captured as well. We acknowledge that this analytic plan may change significantly depending on findings, particularly if there are little observational data regarding the discussion of the disposition decision—although this will be an important finding in and of itself.

#### Rigor and Trustworthiness

Multiple elements of this study design contribute to its rigor and trustworthiness [30]. To maintain credibility, we acknowledge the personal experiences of the researchers performing data collection and analysis as described above. In this case, using more than one coder increases study rigor and will allow for input by someone immersed in the ED environment as a clinician, as well as someone with a nonclinical perspective. This study also uses multiple data sources with observations and interviews to verify findings. We acknowledge that the selected patient population of veterans may limit the transferability, and this is to be further discussed as a limitation. To enhance dependability, we have created a detailed standard operating procedures document with changes documented. To address confirmability, we are creating postobservation reflections after each observation to document evolving thoughts during the research process, and this will also serve as a reference as needed to inform data analysis for each unit.

#### Patient and Public Involvement

Two sources of patient and public involvement were used in the design of this protocol. This study was funded by the Geriatric Emergency Care Applied Research Network 2.0 – Advancing Dementia Care (GEAR 2.0 ADC), a National Institutes of Health–funded program to support research to improve emergency care for people with dementia [31]. The initial grant application and study design proposal were reviewed by community reviewers involved in the GEAR 2.0 ADC program. Multimedia Appendix 3 presents a summary of peer-review findings. Based on their comments, we elected to make changes to the proposed protocol to expand the sample for the aim 2 interviews to all participants included in aim 1 and to expand the sample of persons living with dementia to all levels of dementia severity. As part of the GEAR 2.0 ADC grant program, we were also given the opportunity to consult with the University of Wisconsin’s Community Advisors for Research Design and Strategies (CARDS) program [32]. This is a group of patient and care partner representatives recruited from community center programs to bring perspectives from diverse racial, socioeconomic, and educational backgrounds to all parts of the research process. While CARDS is not specific to dementia care, for this protocol, we specifically sought input into the ideal timing of when to approach patients and care partners for consent during the course of the ED visit, as well as the design of a recruitment flyer to use when first approaching patients and care partners in the ED.

## Results

Data collection was initiated in October 2023. A total of 576 patients have been screened across 31 days of recruitment in the ED. Of those, 47 had a documented diagnosis of dementia, and 23 of those were eligible for inclusion. A total of 17 dyads were approached for consent. As of July 2024, a total of 13 dyads have been recruited. This study is expected to conclude by December 31, 2024.

## Discussion

### Expected Findings

We have designed this novel study to better understand communication practices for persons living with dementia in the ED. While this study is largely focused on the disposition conversation, using an open-ended direct observation tool to capture the clinical encounter will additionally allow us to collect rich, holistic data about the experience of ED care for persons living with dementia and their care partners. We aim to learn more about how disposition conversations take place in a real-world setting, who participates, and what elements of SDM are used or not used. Further, this open approach to direct observation data collection will also capture any number of possible findings, including the very real possibility that there will be a lack of formal discussion around the disposition decision altogether. Our results will be based on observation notes, observation reflections, and semistructured qualitative interviews. While data collection is ongoing, there have been some early lessons noted that merit discussion.

The research team has identified some early lessons related to the recruitment of patients for direct observation research in the ED setting, particularly that some flexibility is needed in the recruitment protocol. Our goal is to observe as many of the conversations between ED providers, patients, and care partners as possible, ideally including the very first conversation conducted involving introductions. As such, we seek to consent of patients and care partners for participation at the time of their ED visit, but before they are seen by any ED physician provider. An early concentration of planning for this protocol was focused on identifying the ideal timing to approach patients and care partners for consent and assent. Given that ED visits are typically for acute, unplanned care, we anticipate that some patients or care partners will understandably prefer to focus on initiating their medical care with their physician provider before they consider participation in research at the time of their ED visit. Our protocol is designed so that the researcher approaches patients and care partners for consent after they are physically moved to a private ED room and assessed by the assigned ED (nontriage) nurse for that room, but before they are seen by an ED physician provider. This typically represents a short time window and requires flexibility if the workflow changes. Our decision-making around this part of the protocol was informed by (1) the clinical experience of one of the researchers (JS) who is a practicing ED physician and (2) direct input from a patient and care partner advisory board. Overall, our approach is designed to prioritize patient care and safety, minimize intrusiveness to ED workflow, and maximize patient and care partner comfort with the timing of research procedures. However, we recognize that this particular ED workflow process may not be the norm in all EDs. As such, the ideal timing for the identification of eligible patients and the approach for consent may differ in other ED settings.

The research team has also identified several early lessons related to research with persons living with dementia and their care partners, some of which are also specific to the ED setting. For example, our protocol was written with several methods for screening ED patients specifically to identify those with dementia. Based on input from practicing clinicians on the research team, we elected to allow for the identification of a dementia diagnosis from multiple locations in the chart as this best mimics methods by which ED providers would look for a dementia diagnosis in a real ED encounter. This includes looking at the “problem” or “diagnosis” list on the patient’s EMR cover sheet, using the medication list, looking at recent outpatient clinic visits or hospital discharge notes, and using the EMR search function. From early data collection, we have identified that most patients will have a diagnosis listed in outpatient visit notes, but it is not as consistently listed in the formal problem list for the patient. This is not surprising as documentation of a dementia diagnosis is well-known to be inconsistent in administrative data using formal diagnosis codes [33]. As such, research procedures for screening for persons living with dementia in real time in the ED will likely need to vary across different health systems that use other types of EMR programs.

In addition, we have found that the person who serves as the patient’s formal LAR may not always be the same as the care partner at the bedside at the time of the ED visit. We anticipated that this may be a more likely scenario compared with other health care settings, given that ED visits are typically unscheduled, and so the patient’s LAR may not be available at the time due to other commitments or having other reasons limiting their ability to attend an unplanned ED visit. As such, we created our protocol to accommodate a variety of consent and assent procedures with this in mind. From early data collection, we have found a variety of people serving as the care partners at the bedside—the majority have been spouses, but also have encountered the patient’s children, in-laws, or family friends. Other studies involving persons living with dementia requiring consent and assent at the time of an ED visit will likely need to include similar approaches.

### Limitations

We also note our study will have several anticipated limitations. As it takes place in a Veterans Affairs setting, this may limit generalizability. Given the demographic makeup of older veterans, we anticipate having predominantly male patients with (female) spousal care partners. We also note that the DVAMC has selected programs aimed at improving the coordination of care for community-dwelling veterans living with dementia, and patients participating in these programs may have access to specific resources that would impact the disposition decision [34]. Our protocol is also focused on capturing communication between patients, care partners, and ED physician providers, but we will not include observations of conversations with specialists consulted by the ED physician, which may mean we miss information conveyed to patients or care partners if the ED provider is not present. We also acknowledge that for this pilot study, patient recruitment will be conducted during business hours. This is typically when more hospital resources and consultants are available to ED providers, which may also influence the course of the ED visit and disposition decision.

### Conclusions

Little is understood about how ED disposition conversations take place in a real-world ED setting and what elements of SDM are used (or not used) when caring for persons living with dementia and their care partners. This study uses direct observation methods to capture the entirety of the ED encounter and will be further informed by participant perspectives elicited by follow-up interviews. Results may be used to provide insights on timing, delivery, personnel, and format of a potential future SDM tool or decision aid for this clinical scenario.

## Appendix

Multimedia Appendix 1 Direct observation notes template.

Multimedia Appendix 2 Patient and care partner interview guides.

Multimedia Appendix 3 Peer-review summary comments from The Geriatric Emergency Care Applied Research (GEAR 2.0-ADC) Community Review Committee (National Institutes of Health, USA).

## Abbreviations

CARDS: Community Advisors for Research Design and Strategies
DVAMC: Durham Veterans Affairs Medical Center
ED: emergency department
EMR: electronic medical record
GEAR 2.0 ADC: Geriatric Emergency Care Applied Research Network 2.0 – Advancing Dementia Care
LAR: legally authorized representative
ODSF: Ottawa Decision Support Framework
SDM: shared decision-making

## Data Availability

The datasets generated and analyzed during this study are not publicly available due to the nature of the research containing potentially identifying and sensitive patient information.
