# Supplementary material for: Characterizing Emergency Department Disposition Conversations for Persons Living With Dementia: Protocol for an Ethnographic Study
Source: JMIR Res Protoc. 2024 Dec 6;13:e65043. doi: 10.2196/65043 (PMC11662188; doi:10.2196/65043)
Supplement: Multimedia Appendix 1 [file resprot_v13i1e65043_app1.pdf]

Study Title: Characterizing ED Disposition Conversations for Veterans with Dementia Using Direct Observations
PI: Seidenfeld, Justine

Date: ______	Time start/stop: ______	Patient ID#: ______	Care partner ID#:______
Recorder: ______	Interaction #: ______	Location: ______	Provider type: ______

Shared Decision-Making for Emergency Department Disposition

Observation Tool*: to be used during interactions between Veterans, care partners and ED providers to capture what happens when they discuss the decision to admit a patient to the hospital or discharge to home. Enter electronically upon return so it can be coded.*

Observations and descriptive field notes

Write linear, descriptive fieldnotes, including timing, who was there, the general content and flow of the conversation, what is happening around them, overall engagement of participants. Can include conversation contents, non-verbal elements, paraphrased quotes, open ended descriptive field notes. Any analytic notes that interpret what is being observed (e.g., that the care partner seems frustrated) should be clearly identified with [closed brackets] to identify this as distinct from the descriptive data.

Observations and descriptive field notes, continued:

Shared Decision-Making Support Features *(based on the Ottawa Decision Support Framework)*
Check off features of shared decision-making support observed during the interactions, and make notes/reference to any data in the field notes on previous pages that relate to these features. See definitions/examples on the next page. Indicate if any of these seemed to play a greater role in guiding the decision-making process.

☐ 1: Establishing rapport and facilitating interactive communication:

☐ 2: Clarifying the decision to be made and inviting participation:

☐ 3: Assessing decisional needs:

☐ 4a: Facilitating receptivity to information:

☐ 4b: Providing information and verifying understanding:

☐ 4c: Clarifying personal values:

☐ 4d: Discussing decisional roles:

☐ 4e: Supporting deliberation and mobilizing resources:

☐ 4f: Facilitating progress in decision making stages:

Shared Decision-Making for Emergency Department Disposition

Domain Definitions – Based on the Ottawa Decision Support Framework

1: Establishing rapport and facilitating interactive communication

- Demonstrating back-and-forth conversation
- Invites the patient/care partner to share, participate, or respond
- Provider demonstrates listening

2: Clarifying the decision to be made and inviting participation

- Provider frames the disposition as a choice to be made
- The provider also invites or encourages the patient/care partner to give input to help make the decision

3: Assessing decisional needs

- Asking what the patient/care partner needs to know to make the decision
- Encourages additional questions from the patient

4a: Facilitating receptivity to information

- Assess openness to making a choice versus already having a decision make
- Check understanding of essential facts
- Allows for time to process new information

4b: Providing information and verifying understanding

- Provider shares pros and cons of admission or discharge, tailored to the individual patient cand care partner
- Provider describes what information (e.g. lab tests, consults) can be used to help make a decision
- Asking questions to verify patient/care partner knowledge and understanding to fill in any gaps or questions.

4c: Clarifying personal values

- Ask about patient/care partner preferences or explicitly invite them to share their views
- Ask about the personal importance of the different options (admission vs discharge)
- Discussing what matters most
- “If X is most important, I would/patients usually choose A; if Y is most important, I would/patients usually choose B”

4d: Discussing decisional roles

- Clarify who is contributing to the decision
- Ask if they would like others to be involved in this decision
- Ask if the patient / care partner wants to be involved in the decision

4e: Supporting deliberation and mobilizing resources

- Giving the patient / care partner time to decide if needed
- Attending to psychosocial issues or resources that might influence the decision

4f: Facilitating progress in decision making stages

- Asking open-ended questions to move the discussion
- Clarifying any remaining questions as new data influences the decision
