# Supplementary material for: Characterizing Emergency Department Disposition Conversations for Persons Living With Dementia: Protocol for an Ethnographic Study
Source: JMIR Res Protoc. 2024 Dec 6;13:e65043. doi: 10.2196/65043 (PMC11662188; doi:10.2196/65043)
Supplement: Multimedia Appendix 2 [file resprot_v13i1e65043_app2.pdf]

PLWD Aim 2 Semi-Structured Interview Guide

Thank you for being here today to participate in this study. Let's introduce ourselves. My name is [name]. I am a researcher with the Durham VA, and I will be interviewing you. What is your first name? ______ So nice to meet you.

This interview is to understand the experiences of persons with memory problems. We especially want to learn what it was like for you when you were in the emergency department [___number of days ago___]. We are interested what happened when you and [___care partner name___] talked to the doctor about whether you should be admitted to the hospital or should go home. We know you were [admitted/discharged]. We want to understand what the experience was like for you so we can try to improve the way health care is provided. We'll be interviewing [___care partner name___] separately to understand their experiences.]

There are no right or wrong answers. You are the expert and we value the unique information that you can tell me about your experiences.

You can choose not to answer a question or you can stop participating at any time.

The interview should take us around 30-45 minutes. You will receive $50 for participating.

Do you have any questions for me so far?

[If yes, answer the participant's questions.]

As noted when we consented you for this interview during your ED visit, this will be audio recorded. Is this still ok? **[Begin audio-recording or choose to take notes if refuses at this late stage, <u>don't</u> lose the interview)**

I am turning on the recorder

Let's begin.

******

- First off, how are you doing today?

- To start with, tell me about your emergency department visit. What happened to you that made it necessary to go to the emergency department?
    - Probe: what was your goal in going to the emergency department? Was that the same or different than [___care partner___]?

- What else happened during the emergency department visit?
    - Probe: Did you have any blood tests or imaging tests done?

PLWD Aim 2 Semi-Structured Interview Guide

Now I am going to ask some questions about what happened at the end of your emergency department visit.

At the end of the emergency department visit, you were [admitted to the hospital/ discharged to go back home].

- How did you feel about that decision? Would you have preferred [the alternative]?
    - Probe: Why?

- How did your doctor or doctors talk to you about that decision, to stay in the hospital or go home?
    - Probe: Did you talk to more than one person about it?

[Establishing rapport]
- Did you feel like you were able to have a back-and-forth conversation with your doctor about it?
    - Probe: Do you feel like your doctor was paying attention to what you had to say? Probe: What could they have done to make you feel more comfortable with sharing or participating?

[Clarifying the decision]
- Did your doctor explain that there was a decision to be made about this?
    - Probe: Did you get the sense that there were different possible options for you? Or did it just seem like "we're going to do this one".

[Decisional needs]
- What kind of information that you got during the emergency department visit helped you in figuring out for yourself if you wanted to go home or stay in the hospital longer?
    - What other kinds of information would have helped you to make a decision about that?

[Facilitating receptivity to information]
- Before you even got to the emergency department, did you think you needed to be admitted to the hospital, or were you hoping to go home after they helped you?
    - [If the opposite happened] What happened to help change your mind about it?
- Do you know if [___care partner___] wanted the same thing? Why do you think they wanted that?

[Providing information]
- What did the doctor say about the pros and cons of [their disposition]? What did they say about [the alternative]?
- How much new information did they give you during the emergency department visit?
    - Probe: did it feel like too much or too little?
- Was there anything that you wanted them to explain more about?
    - Probe: did you ask them? (why or why not?)
    - Probe: did they explain it more? Did that help you? If so, how?

PLWD Aim 2 Semi-Structured Interview Guide

[Personal values]

- For yourself, what were the most important things that made you want to [go home or be admitted].
  - Probe: was there something you were going to worry about or miss if you did or did not go home?
- Did the doctor ask you or talk to you about those things specifically?
  - Probe: Do you think they considered those things when they talked about the options with you?
- What did you think was most important for [____care partner___]?
  - If different: Probe- what did you think about that?

[Decisional roles]

- Did you feel like it was your decision to make?
  - Probe: What made it harder for you to participate? What would have made it easier?
- Did [____care partner____] have a role in that decision?
- Was there anyone else you would have liked to have involved in that conversation?

[Supporting deliberation/facilitating progress]

- Did you feel like you had enough time to make the decision?
- Did it feel rushed?
- Were there other people in the emergency department that helped you with the decision?
  - Probe: like a physical therapist, or a social worker who could help you get more assistance at home?
- Did you get all of your questions answered before the decision was made?

Is there anything else you would like to add or share with me about your visit to the emergency department?

Thank you. **I am turning off the recorder.** I want to thank you so much for all of the valuable information you shared with me today, and we very much appreciate your time.

[For the first few enrolled patients, a few logistic follow-up questions may be asked to help improve the interview flow for subsequent participants. These include asking about topics such as:

- *What was confusing about this interview, if anything?*
- *What could have been explained more clearly?*
- *Did you lose track of what I was asking you at any time? If possible, please describe when.*
- *Did you feel bored or impatient? If possible, describe when you felt this way.]*

Are you interested in receiving a summary of the study findings?

Are you ok with us contacting you again in case we have to ask a clarifying question?

You will receive a check from the Durham VA to compensate you for your time today.

Thank you again for your time today!

INTERVIEW QUESTIONS: Care partner of person with memory problems

Thank you for being here today to participate in this study. Let's introduce ourselves. My name is [name]. I am a researcher with the Durham VA Medical Center, and I will be leading the interview. [*If applicable: [Name] is also a researcher with me at the Durham VA, and he/she will assist with taking notes.]* As discussed in the consent process, we plan to record this interview.

The purpose of the interview is to understand your experiences being the care partner of with a person with memory problems. In particular, what it was like for you when [____patient____] was in the emergency department [___number of days ago___]. We are interested what happened when you and [___patient___] talked to the doctor about whether [he/she] should be admitted to the hospital or should go home.

There are no right or wrong answers to the questions I'll ask, only opinions. Please feel free to share your candid thoughts. You are the expert here, and there is no one else we can ask to get the unique information that you can tell me about your experiences.

We plan to audio record the interview because I want to make sure I don't miss any of your comments. *[If refused at last minute, take detailed notes during the interview instead. Do not lose the interview]*. The recording will be stored securely at the Durham VA and eventually destroyed after we publish the study's findings.

Please know that participating in this interview is voluntary. You can choose not to answer a question or you can stop participating at any time.

You will receive $50 for taking part in today's discussion. Compensation will be provided to you soon after this discussion, in the form of a check mailed to your address [*be sure this information is collected*].

The interview should take us around 30-45 minutes. Do you have any questions for me so far about the interview?

[If yes, answer the participant's questions.]

Are you okay with our conversation being audio recorded? **[Begin audio-record if so, take notes if not]**

Let's begin.

Warm up (consider some of these rapport-building opening questions)

- How do you know [___patient___]? How long have you known each other?
  - If applicable: How did you first meet?
  - If applicable: How often do you see each other?
  - If a spouse: Do you have any children and/or grandchildren?

- What kind of things do you usually help them with at home?

Spouse version of ED experience and disposition decision

- To start with, tell me about your emergency department visit. What happened with [____patient____] that made it necessary to go to the emergency department?
  - Probe: what was your goal in going to the emergency department? Was that the same or different than [____patient____]?

- What else happened during the emergency department visit?

- Probe: Did [____patient____] have any blood tests or imaging tests done?

Now I am going to ask some questions about what happened at the end of the emergency department visit.
At the end of the emergency department visit, [____patient____] was [admitted to the hospital/ discharged to go back home].

- How did you feel about that decision? Would you have preferred [the alternative]?
  - Probe: Why?

- How did the doctor or doctors talk to you about that decision, to stay in the hospital or go home?
  - Probe: Did you talk to more than one person about it?

[Establishing rapport]

- Did you feel like you were able to have a back-and-forth conversation with the doctor about it?
  - Probe: Do you feel like the doctor was paying attention to what you had to say? How about [____patient____]?
  - Probe: What could they have done to make you feel more comfortable with sharing or participating?

[Clarifying the decision]

- Did the doctor explain that there was a decision to be made about this?
  - Probe: Did you get the sense that there were different possible options for [____patient____]??

[Decisional needs]

- What kind of information that you got during the emergency department visit helped you in figuring out for yourself if you wanted [____patient____] to go home or stay in the hospital longer?
  - What other kinds of information would have helped you to make a decision about that?

[Facilitating receptivity to information]

- Before you even got to the emergency department, did you think [____patient____] needed to be admitted to the hospital, or were you hoping to go home after they helped [____patient____]??
  - [If the opposite happened] What happened to help change your mind about it?
- Do you know if [____patient____] wanted the same thing? Why do you think they wanted that?

[Providing information]

- What did the doctor say about the pros and cons of [their disposition]? What did they say about [the alternative]?
- How much new information did they give you during the emergency department visit?
  - Probe: did it feel like too much or too little?
- Was there anything that you wanted them to explain more about?
  - Probe: did you ask them? (why or why not?)
  - Probe: did they explain it more? Did that help you? If so, how?

[Personal values]

- For yourself, what were the most important things that made you want [____patient____] to [go home or be admitted].
  - Probe: was there something you were going to worry about if [____patient____] did or did not go home?
- Did the doctor ask you or talk to you about those things specifically?
  - Probe: Do you think they considered those things when they talked about the options with you?
- What did you think was most important for [____patient____]?
  - If different: Probe- what did you think about that?

[Decisional roles]

- Did you feel like it was your decision to make?
    - Probe: What made it harder for you to participate? What would have made it easier?
- Did [____patient____] have a role in that decision?
- Was there anyone else you would have liked to have involved in that conversation?

[Supporting deliberation/facilitating progress]

- Did you feel like you had enough time to make the decision?
- Did it feel rushed?
- Were there other people in the emergency department that helped you with the decision?
    - Probe: like a physical therapist, or a social worker who could help you get more assistance at home?
- Did you get all of your questions answered before the decision was made?

If you have other thoughts to share before we end the discussion regarding that visit to the emergency department, we would enjoy hearing them.

Thank you. **I am now turning off the recorder**

[For the first few enrolled patients, a few logistic follow-up questions may be asked to help improve the interview flow for subsequent participants. These include asking about topics such as:

- *What was confusing about this interview, if anything?*
- *What could have been explained more clearly?*
- *Did you lose track of what I was asking you at any time? If possible, please describe when.*
- *Did you feel bored or impatient? If possible, describe when you felt this way.]*

Are you interested in receiving a summary of the study findings?
Are you ok with us contacting you again in case we have to ask a clarifying question?
Both you and [____patient____] will receive a check from the Durham VA to compensate you for your time for participating.
Thank you for your time today!
