# Supplementary material for: Characterizing Emergency Department Disposition Conversations for Persons Living With Dementia: Protocol for an Ethnographic Study
Source: JMIR Res Protoc. 2024 Dec 6;13:e65043. doi: 10.2196/65043 (PMC11662188; doi:10.2196/65043)
Supplement: Multimedia Appendix 3 [file resprot_v13i1e65043_app3.pdf]

GEAR 2.0-ADC COMMUNITY REVIEW COMMITTEE APPLICATION REVIEW
2023 - 2024 GRANT CYCLE

APPLICATION NUMBER: EMF22_GEAR_00006

APPLICATION TITLE: Characterizing Emergency Department Disposition Conversations for Veterans with Dementia Using Direct Observations

APPLICANT NAME: Justine Seidenfeld, MD

APPLICANT INSTITUTION: Durham VA Medical Center

Please note that grant scoring follows the NIH scale.

Scientific Review Overall Score (M): 4.05

Community Review Overall Score (M): 2.13

INVESTIGATOR'S STATEMENT OF HYPOTHESIS AND/OR OBJECTIVES OF PROPOSED RESEARCH (VERBATIM)

For Veteran persons living with dementia (PLWDs), one of the most impactful and costly elements of emergency department (ED) care is the decision to discharge or admit them to the hospital- the "disposition" decision. For those requiring urgent treatment in the hospital, the decision to admit is straightforward; but in many cases, this decision is not so simple.[1] In these cases, ED providers must balance the risks of unnecessary admissions, such as delirium and functional decline, against the risks of discharge, which may result in return ED visits and hospitalizations. This decision is especially complex for PLWDs, as they have higher risks of these adverse events compared to their counterparts without dementia.[2-5]

When more than one reasonable option exists regarding a health care decision, such as the decision to admit or not, shared decision making (SDM) can facilitate conversations between patients, care partners, and providers, and help patients make decisions based on their goals, values, and preferences.[6,7] Patient and care partner involvement in SDM can take a variety of forms, with varied degrees of "sharing" in the final decision. It is increasingly recognized that providers underestimate the degree to which patients and care partners want to be involved.[8] However, even compared to older adults without dementia, data on best practices in ED communication and SDM for PLWDs and their care partners are limited.[9] The disposition decision is a crucial area for improving communication and SDM. In a study of ED patients who could recall being involved in any type of SDM during their visit, the "admission versus discharge" decision was the most common, cited by nearly 1 in 5 participants.[10] Furthermore, at the Society for Academic Emergency Medicine (SAEM) Consensus Conference that focused on SDM, decision making regarding hospitalization of older adults was identified as a priority area for further research.[11]

However, effective interventions require real world data about the context within which they are implemented. To determine the optimal timing for an SDM tool, we need foundational data on current communication practices for disposition discussions (e.g., is it addressed across multiple interactions over the course of an ED visit, or mostly at the very end?). To best facilitate participation from PLWDs and care partners, we need to understand what kind of decisional roles they each tend to take. To improve the degree of "sharing" during these discussions, we need to understand barriers and facilitators to participating in an SDM conversation around disposition. However, these complex issues cannot be wholly captured with surveys or participant recall. To address these gaps, the primary objective of this proposal is to use direct observation methods to characterize current practices in disposition conversations for Veteran PLWDs. Direct observation methods are underutilized in EM research but can provide unique and nuanced information about healthcare processes and behavior that may not be captured by self-reported means.[12] This work will be used to inform a future VA-NIA Career Development Award to develop an SDM tool to support disposition decisions for PLWDs and care partners. In collaboration with geriatric, nursing, and health services research colleagues, I propose a pilot study with the following aims:

Aim 1: Characterize discussions about ED disposition with PLWDs and their care partners. Approach: Using direct observations methods during ED visits for n=20 PLWDs with

care partners present, we will characterize timing, decisional roles, and decision support strategies used in disposition conversations. Strategies used for decision support will be characterized using the Ottawa Decision Support Framework (e.g., establishing rapport, inviting participation, clarifying personal values, supporting deliberation).[13] The degree of patient and care partner involvement in the decision will additionally be evaluated using the observer-rated OPTION-5 scale.[14] To ensure that we include PLWDs identified by the SAEM Consensus Conference on SDM as those with healthcare disparities that may experience an SDM intervention differently, we will deliberately sample from 1) racial and ethnic minority groups, 2) those with severe dementia, and 3) those with low health literacy.[15,16] Rationale: identifying current practices in ED disposition discussion with PLWDs and their care partners will inform the timing, format, and decision support elements of a future SDM tool.

Aim 2: Identify facilitators and barriers to participating in disposition decision making for PLWDs and care partners. Approach: Based on OPTION-5 scores and findings from direct observations in Aim 1, we will conduct semi-structured qualitative interviews with a subset of Aim 1 participants- 4 PLWD and care partner dyads (n = 4-8 interviews based on PLWD participation) with notably *high* engagement and 4 PLWD and care partner dyads (n = 4-8 interviews based on PLWD participation) with notably *low* engagement in the disposition discussion. We will use interviews to ask participants about specific observations made from their encounter, to gain insight into their perspective on their role and elements of decision support used during that conversation. Rationale: this will inform tailoring of a future SDM tool to ensure it can incorporate facilitators and address any common barriers to engagement in a disposition discussion.

The expected outcomes of this study are to identify current practices, barriers, and facilitators for ED disposition decision making conversations with PLWDs and their care partners. This will have a positive impact on the experience of ED care delivery for Veteran PLWDs and their care partners, as it will be used to inform optimal timing for use of an SDM tool, elements of decision support that must be included, and how to ensure that it will work for subgroups of PLWDs who may have communication- or SDM- related healthcare disparities.

SCIENTIFIC REVIEW COMMITTEE

GRANT DISCUSSION SUMMARY

There was strong support for this grant amongst reviewers. Reviewers did have concerns that the approach is more ethnographic rather than content based. If the content is the interest, then reviewers recommend using an audio recording model without the prescreening. Reviewers noted much of the discussion about disposition is set up in the first few minutes of the interaction, which will be challenging based on this protocol. Additionally, reviewers noted that narrowing the process to a few key diagnoses would be more powerful (e.g., chest pain) rather than the current screening process. Finally, reviewers felt it was unclear how patients with delirium will be excluded based on the currently described methodology.

OVERALL IMPACT SUMMARY

Primary Reviewer
The applicant is a well-trained junior researcher who proposes to work in the VA system to examine the disposition decision of PLWD. She argues that the burden of admission is significant, and in cases of clinical equipoise the use of SDM approaches would have value. She proposes to evaluate this by observing ED care and conversations regarding the disposition decision making and interviewing the PLWD and care partner. While important (disposition), it is not clear that the SDM approach is optimal or that the approach is sufficient particularly related to inclusion criteria and sample size. Finally, there is a large number of individuals listed as part of this study, which will be challenging to manage.

Secondary Reviewer
This is a strong proposal focused on using direct observation methods to characterize current practices in ED disposition conversations for veterans with dementia and caregivers to inform future development of a shared decision-making tool. There is a clear need for available tools to promote SDM conversations about the decision regarding hospitalization in the ED for patients with dementia. The study team and environment are ideally suited to carry out the proposed project. The project appears feasible and has a clear timeline for providing outcomes to inform the next phase of the research. Minor weaknesses regarding lack of information about the racial/ethnic minority groups to be recruited and potential bias in data collection are noted. Overall, the impact of this proposal is rated as high.

SIGNIFICANCE SUMMARY

Primary Reviewer
The overall goal is to create a shared decision-making tool to decide on admission vs discharge for PLWD. First is to know how the disposition discussion currently takes place, which this study proposes to do by observing the discussion and then interviewing the patients / care partners regarding their engagement.

While whether the patient should be admitted or not is an important question, it is not clear the SDM model and approach are correct. This model can work when we have clinical equipoise, but in the PLWD goals of care are separate but more important than SDM.

One question that is not clear to me: given that the current conversation is likely not optimal, does it matter what currently happens?

Secondary Reviewer
Results from this study will contribute to understanding of barriers and facilitators to shared decision making in ED disposition discussions. This information will inform optimal timing for use of an SDM tool, elements of decision support that must be included, and how to ensure that it will work for subgroups of PLWDs with healthcare disparities. These results will inform development of an SDM tool to support disposition decisions for people with dementia and care partners in a future planned VA Career Development Award application.

INVESTIGATORS SUMMARY

Primary Reviewer
The applicant is well trained, having completed the NCSP and working within the VA under the guidance and mentorship of excellent experts with both care transitions and geriatrics expertise. She has experience in qualitative research and working within the VA system.
Dr. Sperber will be an important investigator, as her qualitative expertise is critical.

Secondary Reviewer

- Investigator and mentorship team has significant relevant experience in researching geriatric ED populations, persons with dementia and caregivers, and qualitative methodology.
- PI has excellent prior relevant clinical research experience to support this project including several in-press publications in this area.
- Roles of study team are clearly defined.

INNOVATION SUMMARY

Primary Reviewer
While direct observations have not been often done with PLWD, they are done in the ED as it is a valuable method.

The use of the Ottawa framework is novel.

Secondary Reviewer

- This would be the first use of direct observation methods with PLWDs in the ED setting (vs interview, survey, focus groups, or video recordings) which may provide richer information about complex processes.
- Application of an existing conceptual framework in a novel way (for dementia decision-making in the ED).

APPROACH SUMMARY

Primary Reviewer

For aim 1, the applicant proposes complete observation of the entire ED visit by patients who are known to have dementia. During this visit, the PI and a staff member will take notes to record all aspects of the visit that has to do with disposition decision making. There are a number of challenges to what they propose. First, there is an Issue regarding numbers, as they propose 20 participant cases in aim 1, but have 6different permutations of characteristics, leaving only about 3 per category. Second, there is also an issue regarding the cases chosen for observation. The applicant proposes to choose cases by asking the physician to determine, based on the triage note, whether there is the potential for disposition decision making. However, this will likely result in the inclusion of numerous irrelevant cases based on the lack of ambiguity related to SDM. I am concerned that the decision on sample size is not clearly considered.

For aim 2, the approach is generally solid, with interviews occurring a few days after ED care is complete. My primary concern with this aim is the sample size determination. The PI is fixed on performing interviews with 8 of the 20 included, but there is no consideration of whether these interviews will be truly representative.

The applicant has considered a number of potential issues with the study and has considered approaches to address the challenges.

Secondary Reviewer

Strengths

- Provides information on preliminary data to support recruitment of Veterans with dementia and caregivers.
- Will consult with stakeholder group (VetREP) to ensure that participant-facing materials are patient-centered, literacy-tailored, culturally appropriate, and feasible for patients with more advanced dementia as much as possible.
- Includes thoughtful design challenges and potential solutions, as well as clear study timeline and next steps.

Weaknesses

- Would be helpful to know how many patients with dementia with caregivers are seen in the setting ED. Is there concern about excluding those with healthcare disparities based on eligibility criteria requiring caregiver present (e.g., are those patients less likely to present with caregivers)?
- Lack of information about specific types of racial/ethnic minority groups targeted for this study, how many participants from racial/ethnic groups will be targeted, and any culturally-specific approaches used for recruitment, enrollment, or assessment of patients from racial/ethnic minority groups will be used. For example, will there be a study team member from a racial/ethnic minority group to conduct observations to reduce potential racial bias in data collection or gather information from bilingual patients? If not, what will the limitations to the results be? Also, how many patients from racial/ethnic minority populations of interest are typically seen in the ED?

ENVIRONMENT SUMMARY

Primary Reviewer
The applicant is leveraging resources at the VA, including geriatrics, the COIN, and research expertise.

Secondary Reviewer
Resources, facilities, and equipment are appropriate for the needs of the proposed project.

COMMUNITY REVIEW COMMITTEE

GRANT DISCUSSION SUMMARY

Reviewers felt this was a strong proposal and liked the fact that it worked within the VA system. They liked the focus on shared decision-making and how it would work to improve dyad communication, but many reviewers were concerned about limiting the sample to severe dementia as valuable insight could be derived from individuals in earlier stages of the disease, from younger individuals living with the disease, and/or those coming from a more diverse background. Generally, not looking into some of the sub-groups impacted by dementia could be a missed opportunity. They also wanted to see more inclusion of care partners and clinicians in understanding the communication practices overall.

RELEVANCE TO EMERGENCY CARE OF PLWD SUMMARY

This protocol addresses the GEAR 2.0-ADC ED Care Practices, Communication and Shared Decision-Making, and ED Care Transitions priorities. Reviewers were a bit unclear what the shared interaction between the ED staff and the dyads (care partners and PLWD) would be and felt a clearer engagement plan could be useful. Some suggested also capturing data from the clinician's point of view to enhance this point.

FEASIBILITY SUMMARY

Reviewers felt the study had high feasibility, but they also encouraged the applicant to seek out more PLWD voices to provide insight during study development. Reviewers recommended this based on some of the applicant's written uncertainties for having valuable discussions with PLWD.

HEALTH EQUITY SUMMARY

Reviewers noted the project recognized cultural views and did a good job of incorporating steps in recruitment based on race and literacy. Some reviewers wanted to see a bigger expansion of dementia groups to include mild and moderate cases, as they worry the severity cutoff could be restrictive.

PROTECTIONS SUMMARY

Reviewers noted that data security and population sensitivities are well accounted for, and its clear the protocol is minimal risk.
